# Ten rules to assess and manage the acutely deteriorating patient: a practical mnemonic

**DOI:** 10.1186/1754-9493-5-29

**Published:** 2011-11-15

**Authors:** Katherine M Baugher, Amal Mattu

**Affiliations:** 1 Department of Emergency Medicine, University of Maryland School of Medicine, 110 S. Paca Street, 6th Floor, Suite 200, Baltimore, MD 21201, USA

**Keywords:** ABC's, Cardiac arrest, Compressions, Crashing, Defibrillation, Resuscitation

## Abstract

The acutely deteriorating patient is a challenge to even the most seasoned provider. The ability to diagnosis the underlying condition quickly and accurately is vital to a successful outcome. We present a review of 10 critical aspects in the management of the crashing patient, based on up-to-date guidelines and organized as an easily remembered mnemonic. The A-A-B-B-C-C-D-D-E-E's of the deteriorating patient address many key pearls and current recommendations to give physicians an added advantage in the moment of crisis.

## Introduction

Each year in the United States, an estimated 295,000 people experience out-of-hospital cardiac arrest [1] and another 200,000 experience in-hospital cardiac arrest [2]. Additionally, hospital care providers frequently encounter patients who were previously stable but have begun to rapidly decline. Appropriate awareness and management of peri-arrest characteristics may thwart impending deterioration. Among adults, the most frequent causes of cardiac arrest are cardiac arrhythmia, acute respiratory failure, and hypotension [3]. The unstable, crashing patient requires quick and accurate assessment for appropriate interventions, and the true difficulty in managing this population is reflected by the dismal survival rates. The rate of survival to discharge is higher for in-hospital arrests (21%) [4] than for out-of-hospital arrests (7.9%) [1]. Difficulties arise primarily from the brevity of time available in which to determine the diagnosis. Expending resources and time in pursuit of a misdiagnosis will further harm an already unstable patient. To improve physician response and competence in crisis, we present an easily remembered mnemonic, the A-A-B-B-C-C-D-D-E-E's in the crashing patient.

**Case scenario: **A 55-year-old male who is "not feeling well" is brought to the emergency department by paramedics. He is diaphoretic but awake and alert and is able to speak clearly. On review of vital signs, he is found to be afebrile; tachycardic, with a heart rate in the 120 s; tachypenic, to 28 beats/min; and hypotensive (80/40 mm Hg). The pulse oximetry reading is > 96% and the fingerstick glucose level is 120 dL/mg. An intravenous (IV) line has been established, and the patient is receiving oxygen and is connected to a cardiac monitor. The electrocardiogram (ECG) is non-diagnostic. The patient continues to decompensate, the immediate cause of which is unclear. What should be your early considerations for potential diagnosis and intervention in the next minutes?

*The clinician should consider the A-A-B-B-C-C-D-D-E-E's in this "crashing" patient*.

### A - Aortic Disasters

*Consider abdominal aortic aneurysm (AAA) and thoracic aortic dissection (TAD) for every crashing or arresting patient, regardless of the presence or absence of "typical" symptoms*.

Some of the classic symptoms of AAA (pulsatile abdominal mass, hypotension, and back/flank/abdominal pain) and TAD (abrupt onset of tearing/ripping chest pain radiating to the back) are not present in the majority of cases. In a retrospective study of an Austrian registry of cardiac arrest patients, 50% of the patients who presented with pulseless electrical activity (PEA) arrest from AAA or TAD did not have abdominal/flank pain or abrupt onset of chest pain, respectively, prior to arrest [5]. Similarly, a review of the International Registry of Acute Aortic Dissection revealed that hypotension was present in only about a third of patients with dissection [6]. These studies demonstrate that you cannot rely on a typical presentation to alert you of an aortic disaster.

Although PEA arrest usually brings to mind myocardial infarction or pulmonary embolus (PE), give serious consideration to the possibility of AAA or TAD. Although PEA is often considered a common arrest rhythm in cases of massive pulmonary embolism (PE) and massive myocardial infarction (MI), PEA is the most common cardiac arrest rhythm of AAA and TAD [5,7]. Caution is warranted when considering the empiric administration of thrombolytics for MI or PE: their use in a patient crashing due to AAA or TAD could be fatal.

Bedside ultrasound is a quick procedure for diagnosing aortic disasters [5,7]. A simple scan of the abdomen may reveal an AAA or fluid within the abdomen and pelvis secondary to rupture. TAD might be visualized as cardiac tamponade resulting from progressive retrograde dissection [3]. Because ultrasound can be conveniently performed during a resuscitation, it is hard to justify not performing this exam.

### A - Acidosis (Metabolic)

**Case Scenario: **A 58-year-old nursing home patient is brought to the emergency department with a decreased level of consciousness. He appears to be dehydrated. Vital signs are notable for fever, hypotension, tachycardia, and tachypnea. Arterial blood gas analysis shows a pH of 7.15, pCO_2 _of 18, and HCO_3 _of 10. You suspect sepsis, and fluids and broad-spectrum antibiotics are initiated. However, the patient starts to have difficulty maintaining his work of breathing and requires emergent intubation. After undergoing intubation with no complication and being placed on standard ventilator settings of 16 breaths/min, a tidal volume of 450 mL, 100% inspired oxygen, and positive end-expiratory pressure of 5 cm, the patient arrests. What went wrong?

*Because of the possibility of acidosis, consider preintubation respiratory rates when intubating a rapidly deteriorating patient*.

In the critically ill patient, metabolic acidosis can lead to compensatory respiratory alkalosis. A typical scenario is the Kussmaul breathing seen in patients with diabetic ketoacidosis. When sedating and paralyzing a patient for intubation, respiratory compensation is lost. If the level of respiratory compensation on the ventilator is not similar to preintubation compensation, acidosis can worsen, resulting in brady-asystolic arrest. These complications can be avoided by determining the respiratory rate immediately before intubation and matching the ventilator respiratory rate. A rate < 30 breaths/minute will avoid auto-PEEP [8]. Careful attention to preintubation respiratory rates in acidotic patients requiring intubation will prevent further complications and even death.

### B - Bagging/Breathing

*Ensure that hyperventilation does not introduce further complications in a crashing patient*.

A common mistake made in managing victims of cardiac arrest is overzealous bag-valve-mask ventilation. Professional rescuers (in both the pre-hospital and in-hospital phases of care) often ventilate patients excessively during cardiopulmonary resuscitation (CPR) [9,10]. Excessive ventilation increases intrathoracic pressure, decreases venous return, decreases cardiac output, decreases coronary blood flow, and can ultimately decrease the likelihood of survival [9]. Aufderheide et al [10], using an animal model, confirmed that hyperventilation decreases coronary perfusion and survival rates. Excessively rapid ventilation in these patients can also produce cerebral vasoconstriction, reducing cerebral perfusion and potential neurologic recovery. Largely for these reasons, the resuscitation guidelines issued by the American Heart Association (AHA) in 2010 recommend delivery of only 8 to 10 breaths per minute (one breath every 6 to 8 seconds) during CPR in order to avoid reduced cardiac output and cerebral perfusion. Even in the patient who is not in full cardiac arrest but is in shock, excessive ventilation reduces venous return and preload, potentially producing further dangerous reductions in blood pressure. Hyperventilation should be avoided in patients in shock or cardiac arrest, but if rapid ventilation is necessary because of metabolic acidosis (see above), every attempt should be made to normalize volume status so as to sustain preload.

### B - Baby on Board

*Consider pregnancy and ruptured ectopic pregnancy in every crashing female of childbearing age (9 to 60 years of age). Appropriate management of ventricular dysrhythmia, positioning during resuscitation, and early perimortem cesarean section are critical for an optimal outcome in the pregnant patient*.

Overreliance on the tachycardic response to intraperitoneal hemorrhage may delay proper diagnosis and treatment of a ruptured ectopic pregnancy. In fact, Snyder reported that 55% of hypotensive patients with ruptured ectopic pregnancies were not tachycardic [11]. For this reason, bedside ultrasound should be utilized liberally in pregnant women with either abdominal pain or vaginal bleeding to evaluate for ectopic pregnancy, regardless of the heart rate.

Nationwide, the incidence of cardiomyopathy-induced deaths of pregnant patients is increasing [12]. Cardiomyopathy increases the arrhythmogenic potential of the heart (increased reentry and mechanical stretch), which, when coupled with normal pregnancy-induced changes (increased heart rate, cardiac output, ectopic beats), may increase the incidence of cardiac events [13,14]. Amiodarone, a common first-choice treatment for ventricular arrhythmia, is actually a class D antiarrhythmic medication and therefore should be avoided whenever possible in pregnant patients. Complications of amiodarone include hypothyroidism/hyperthyroidism, intrauterine growth retardation, bradycardia, and prematurity. Alternative medications to consider in the pregnant patient with ventricular dysrhythmia include lidocaine and procainamide, but keep in mind that these and most other medications used for ventricular dysrhythmias are class C drugs [13].

On the other hand, all of the usual modalities for electrical cardioversion of arrhythmias are generally considered safe in pregnancy. Cardioversion, defibrillation, the use of an automated external defibrillator, and cardiac pacing show no clinically significant evidence of harm to the fetus, regardless of the stage of pregnancy [15]. Very little current passes through the uterus to the fetus. Furthermore, the fetal fibrillation threshold is high, so life-saving electrical modalities should not be withheld from the mother out of concern for the effect of current on the fetus. Care should be taken to remove fetal monitors before administering electricity.

The gravid uterus affects patient positioning and the delivery of compressions during resuscitation. During the second half of pregnancy, the uterus can compress the inferior vena cava and impede venous return to the heart when the mother is lying in a supine position. The traditional practice of placing the mother in the left lateral tilt position in order to move the uterus off the inferior vena cava can improve cardiac output by up to 30%. However, good-quality chest compressions are difficult to perform when the victim is in the left lateral tilt position, and only 80% of the normal force of compressions is transmitted in this position [16]. One study [16] showed that by placing the patient in the supine position and having a second rescuer manually deflect the uterus, one could achieve both improved venous return and the optimal force of compressions. The 2010 CPR guidelines published by the American Heart Association AHA have adopted this recommendation as the preferred method of performing CPR during the second half of pregnancy [17]. It is also important to remember that compressions during the second trimester and beyond should be performed more superiorly on the sternum (one to two finger spaces superior) to adjust for the position of the diaphragm and the volume of abdominal contents.

If cardiac arrest persists longer than four minutes or if the mother is obviously fatally injured in the latter half of pregnancy, perimortem cesarean section (PMC) is indicated. PMC not only allows an opportunity to quickly resuscitate the baby, but by removing aorto-caval obstruction, maternal venous return and cardiac output improve as well.

Maternal brain damage is likely after four minutes of cardiac arrest [18]. However, studies show that PMC is frequently performed well after four minutes, despite data supporting that both mother and infant have better outcomes with early PMC [18-20]. As a result of PMC, decompression of the maternal vena cava improves cardiac output and delivery of more effective chest compressions. Studies have shown more than 50% of patients had a dramatic improvement, with return of spontaneous circulation (ROSC) within 15 minutes after PMC [18].

PMC is recommended for any woman at more than 20 weeks' gestation who experiences cardiac arrest [17]. Keep in mind that the gravid uterus can be palpated at the umbilicus at 20 weeks and increases by one centimeter with each additional week. An initial midline vertical incision will expose the rectus abdominis and linea albea. After cutting through the linea alba, a second incision through the uterus allows delivery of the fetus [21]. Performance of PMC confers the best chance of survival for the baby as well as the mother.

### C - Compressions

*Consider evolving trends in the role of chest compressions in the crashing patient*.

A review of 61 out-of-hospital cardiac arrests revealed that chest compressions were performed during only 43% of the time that patients had no pulse [22]. The inadequacy of compressions was attributed largely to numerous sources of interruptions such as bagging, pulse checks, drug administration, and intubation attempts.

The 2010 AHA guidelines recommend that interruptions be absolutely minimized and that continuous end-tidal carbon dioxide (ETCO_2_) monitoring be utilized. Prior to ROSC, ETCO_2 _is likely to be < 10 mm Hg, but at the moment of ROSC, the number can be expected to increase by 35 to 40 mm Hg. The use of ETCO_2 _therefore allows rescuers to detect ROSC without having to interrupt compressions for pulse checks. Caution is advised when administering sodium bicarbonate, because it can falsely elevate these numbers even in the absence of circulation [23].

In addition to limiting interruptions, ensuring a high compression rate has been shown to have a significant positive correlation with initial ROSC [24]. Abella et al [24] reported that more than half of cardiac arrest patients received suboptimal compression rates in their series of 97 cases. As resuscitation recommendations shift the emphasis to increased compression rates, support for cardiocerebral resuscitation (CCR) is growing. CCR represents a paradigm shift from A-B (airway-breathing) to C (circulation) in cardiac arrest victims. Early ventilations are now de-emphasized in primary cardiac arrest patients (excluding those in pulmonary arrest following drowning or opiate overdose, those with life-threatening asthma/COPD, and children in arrest), with an emphasis on passive oxygenation, good compressions at a rate of 100/min, and early defibrillation. Marked increases in survival rates and improved neurologic outcome have been reported with CCR [25,26].

### C - Cooling (Therapeutic Hypothermia)

*Consider the appropriate use of therapeutic cooling*.

Therapeutic hypothermia has proven beneficial in patients who regain spontaneous circulation after experiencing out-of-hospital cardiac arrest caused by ventricular fibrillation [27,28]. The International Liaison Committee on Resuscitation (ILCOR) recommends cooling to 32 to 34 C (90-93 F) for 12 to 24 hours [29]. Cooling can be achieved using simple measures such as application of cooling blankets; administration of 4 C intravenous fluids; and application of ice packs to the groin, axilla, and neck. Therapeutic hypothermia increases the rate of favorable neurologic outcome as well as survival [28]. The 2005 AHA guidelines recommended hypothermia for patients with ROSC after out-of-hospital cardiac arrest whose presenting rhythm was ventricular fibrillation; however, the guidelines also suggested some benefit may be seen with other rhythms or with in-hospital cardiac arrest [30]. Although a recent study found that patients with PEA/asystole actually did slightly worse after induction of hypothermia [31], most recommendations at this time still suggest consideration of therapeutic hypothermia regardless of presenting rhythm, pending additional studies. Its utility in children, pregnant women, and cardiogenic shock patients has yet to be determined.

### D - Decline Position (Trendelenburg)

*Avoid placing a patient in shock in the Trendelenburg position*.

Using the Trendelenburg position to treat hypotension and shock is antiquated and might even be dangerous. This maneuver fails to increase blood pressure and cardiac output in most patients, does not improve tissue oxygenation, results in displacement of only 1.8% of total blood volume, and actually decreases cardiac output in hypotensive patients. Additionally, it produces right ventricular stress and deterioration of pulmonary function [32].

### D - Defibrillation

*Use defibrillation rapidly in the appropriate rhythm*.

If the patient has a shockable rhythm (e.g. ventricular fibrillation, pulseless ventricular tachycardia), rapid defibrillation may be life saving. Monophasic and biphasic waveforms give similar results in terms of ROSC and survival to hospital discharge, although biphasic shocks tend to be successful at lower energy levels than monophasic shocks. Regardless of the type of defibrillator being used, defibrillation should be performed at maximum energy levels when patients are in cardiac arrest (120-200 J for biphasic defibrillators, 360 J for ventricular fibrillation) [33].

Following one shock, immediately administer chest compressions for two minutes before any interruptions to check for a pulse or rhythm. Another way of minimizing interruptions in compressions is to charge the defibrillator during ongoing compressions. When compressions are interrupted to check the rhythm, the defibrillation can be administered immediately if the patient has a shockable rhythm, without any further delay to charge up, and then compressions can be resumed at once.

A relatively new concept of "hands-on defibrillation" has been suggested as yet another way of minimizing interruptions in compressions. Compressions are continued during the delivery of shock. Researchers have found that the current delivered by a biphasic defibrillator cannot be perceived by the person performing the chest compressions [34]. Several of our own colleagues and I (AM) have performed hands-on defibrillation, i.e., biphasic defibrillation during full-force chest compressions, and felt no shock. We anticipate that future studies will provide continued support for this method, and future resuscitation guidelines may incorporate this technique as a valuable way of further minimizing interruptions in chest compressions.

### E - Effusion

**Case Scenario: **A 56-year-old woman with a history of breast cancer presents with chest pain, shortness of breath, fever, tachypnea, and tachycardia. Her blood pressure is stable, her pulse oximetry reading is 96%, her lungs are clear, and she has jugular vein distension (JVD) and a non-diagnostic ECG. What is in your primary diagnosis?

*Consider pulmonary embolism (PE) and as well as pericardial effusion in patients presenting with dyspnea*.

In a patient with pericardial effusion, dyspnea and tachypnea are often caused by mild compression of the bronchial structures by pericardial fluid. Alveolar oxygen exchange, however, is usually unaffected, and therefore oxygen saturation is usually normal. Although the risk factors for PE and pericardial effusion are similar, the treatments are very different, and misdiagnosis can be dangerous. The use of thrombolytics or heparin would be warranted to dissolve the clot of an embolus, but it could result in hemorrhagic effusion and tamponade in a misdiagnosed effusion. Bedside ultrasound can quickly aid in the distinction between these two entities. Pericardial effusions demonstrate fluid around the heart and frequent global hypokinesis. PE, on the other hand, is associated with right ventricular dysfunction and often a hyperdynamic heart.

Large pericardial effusions manifest signs of increased central venous pressure, JVD, ascites, and muffled heart sounds. Acute tamponade is associated with hypotension as well. Although Beck's triad (hypotension, JVD, muffled heart sounds) is a commonly discussed finding in patients with large pericardial effusions or tamponade, only one-third of these patients demonstrate all three findings [35]. Many clinicians associate electrical alternans with large pericardial effusions, but actually it is present in only 20% of patients [36]. In short, the "classic" findings are poorly sensitive or specific, so physicians should have a low threshold to utilize bedside ultrasound to assist with the diagnosis.

In patients who require respiratory support, be wary of noninvasive ventilation and standard mechanical ventilation. Any form of positive-pressure ventilation can have deleterious hemodynamic effects on patients with cardiac tamponade, because of the decrease in preload that positive-pressure ventilation produces [37]. Because patients with large pericardial effusions and tamponade are preload dependent, intravascular volume should be optimized with intravenous fluids to maintain preload. Be prepared for pericardiocentesis if hypotension worsens.

### E - Embolism

*Patients with large pulmonary emboli have unusual hemodynamics. Be wary of administering large volumes of intravenous fluids to these patients when they are hypotensive*.

Patients with massive pulmonary emboli develop obstruction to right-to-left blood flow in the heart. The result is that the right ventricle distends, causing bowing of the interventricular septum into the left ventricle. This reduces left ventricular filling, cardiac output, and blood pressure. Although our normal response to managing hypotension is to aggressively administer intravenous fluids, this actually can be detrimental to the patient who is hypotensive with a massive pulmonary embolus. Fluid boluses further increase right ventricular distension. As a result, the interventricular septum deviates further into the left side of the heart, further decreasing left ventricular filling, cardiac output, and blood pressure. When patients with presumed or confirmed massive PE are hypotensive, use vasopressors early. Thrombolytics in unstable patients with massive pulmonary emboli may restore right ventricular function [38], though definitive data mandating the use of these drugs under these conditions are lacking.

Bedside ultrasound can be helpful in diagnosing massive PE as the cause of hypotension. Right ventricular distension caused by a pulmonary embolus can be detected by practitioners with even basic ultrasound skills. With slightly more advanced skills, bowing of the intraventricular and McConnell's sign (right ventricular free-wall hypokinesis with normal apical contraction) may be seen on ultrasound. These findings are not specific to PE but can also be found in patients with other deadly entities such as right ventricular infarction [39]. The history, physical examination, and ECG should help distinguish among these possibilities.

Hypotension may actually improve after intubation because the increased intrathoracic pressure associated with positive-pressure ventilation reduces preload. The right ventricular distension then decreases, and subsequent bowing of the septum is relieved, increasing cardiac output. New T-wave inversions are often found simultaneously in leads V1 through V3 and inferior leads on the ECGs of patients with large pulmonary emboli. One study identified a key difference in T-wave inversion patterns in PE versus acute coronary syndrome. Simultaneous T-wave inversions in leads III and V1 were far more likely to be associated with PE, whereas the presence of T-wave inversions in I and aVL were almost always acute coronary syndrome [40]. Right heart strain and a tall R wave in V1 also suggest PE.

## Conclusions

The critical moments with a rapidly deteriorating patient should be guided by the A-A-B-B-C-C-D-D-E-E mnemonic (Table 1). Proper use of bedside ultrasound, breathing control, pregnancy considerations, and quality CPR may greatly improve your patients' chances of survival.

**Table 1:** Overview of the A-A-B-B-C-C-D-D-E-E's in the crashing patient.

**A**	**Aortic Disasters**	Do not rely on "typical" symptoms in aortic disasters.
		- Use bedside ultrasound before administering thrombolytics.
**A**	**Acidosis (Metabolic)**	Metabolic acidosis may worsen into bradycardia, asystole, or tachydysrhythmia.
		- Simulate the preintubation rate when setting the ventilation respiratory rate.
**B**	**Bagging/Breathing**	Hyperventilation may decrease survival rate.
		- Ventilate at a frequency no greater than one breath every 6 to 8 seconds.
**B**	**Baby on Board**	Consider normal/ruptured ectopic pregnancy in every female of child-bearing age.
		- Manage ventricular dysrhythmia, resuscitation positioning, and perimortem C-section.
**C**	**Compressions**	Limit interruptions and maintain a high rate of quality compressions.
**C**	**Cooling (Therapeutic Hypothermia)**	Use cooling in unresponsive arresting patients with ROSC.
**D**	**Decline Position (Trendelenburg)**	Avoid using the Trendelenburg position for shock.
**D**	**Defibrillation**	Use defibrillation early, if indicated.
		- Use a single biphasic shock and "hands-on" defibrillation.
**E**	**Effusion**	Thrombolytics can worsen a preexisting effusion.
		- Utilize bedside ultrasound and administer intravenous fluids judiciously.
**E**	**Embolism**	Right heart strain can be made worse with intravenous fluids.
		- Utilize bedside ultrasound and limit intravenous fluids.

## List of abbreviations

ECG: electrocardiogram; AAA: abdominal aortic aneurysm; TAD: thoracic aortic dissection; PEA: pulseless electrical activity; MI: myocardial infarction; PE: pulmonary embolus; CPR: cardiopulmonary resuscitation; PMC: perimortem cesarean section; ROSC: return of spontaneous circulation; ETCO_2_: end-tidal carbon dioxide; IV: intravenous.

## Competing interests

The authors declare that they have no competing interests.

## Authors' contributions

Both authors participated in the preparation of the manuscript and read and approved the final version.

## Authors' information

KB is a second-year emergency medicine resident at the University of Maryland Medical Center. AM is a professor of emergency medicine and vice chairman of the Department of Emergency Medicine at the University of Maryland School of Medicine.

## References

[B1] Nichol G Thomas E Callaway CW Hedges J Powell JL Aufderheide TP Rea T Lowe R Brown T Dreyer J Davis D Idris A Stiell I Resuscitation Outcomes Consortium Investigators Regional variation in out-of-hospital cardiac arrest incidence and outcome JAMA 2008 300 1423 1431 [published correction appears in *JAMA *2008, **300**:1763] 10.1001/jama.300.12.1423 18812533 PMC3187919

[B2] Merchant RM Yang L Becker LB Berg RA Nadkarni V Nichol G Carr BG Mitra N Bradley SM Abella BS Groeneveld PW the American Heart Association Get With The Guidelines-Resuscitation (GWTG-R) Investigators Incidence of treated cardiac arrest in hospitalized patients in the United States Crit Care Med 2011 in press 10.1097/CCM.0b013e3182257459 PMC3196742 21705896

[B3] Peberdy MA Kaye W Ornato JP Larkin GL Nadkarni V Mancini ME Berg RA Nichol G Lane-Trultt T Cardiopulmonary resuscitation of adults in the hospital: a report of 14720 cardiac arrests from the National Registry of Cardiopulmonary Resuscitation Resuscitation 2003 58 3 297 308 10.1016/S0300-9572(03)00215-6 12969608

[B4] Roger VL Go AS Lloyd-Jones DM Adams RJ Berry JD Brown TM Carnethon MR Dai S de Simone G Ford ES Fox CS Fullerton HJ Gillespie C Greenlund KJ Hailpern SM Heit JA Ho PM Howard VJ Kissela BM Kittner SJ Lackland DT Lichtman JH Lisabeth LD Makuc DM Marcus GM Marelli A Matchar DB McDermott MM Meigs JB Moy CS Heart disease and stroke statistics--2011 update: a report from the American Heart Association Circulation 2011 123 4 e18 e209 10.1161/CIR.0b013e3182009701 21160056 PMC4418670

[B5] Meron G Kürkciyan I Sterz F Tobler K Losert H Sedivy R Laggner AN Domanovits H Non-traumatic aortic dissection or rupture as cause of cardiac arrest: presentation and outcome Resuscitation 2004 60 2 143 50 10.1016/j.resuscitation.2003.10.005 15036731

[B6] Tsai TT Bossone E Isselbacher EM Nienaber CA Evangelista A Fang J Smith DE Cooper JV Hutchison S O'Gara P Eagle KA Mehta RH International Registry of Acute Aortic Dissection Clinical characteristics of hypotension in patients with acute aortic dissection Am J Cardiol 2005 95 1 48 52 10.1016/j.amjcard.2004.08.062 15619393

[B7] Pierce LC Courtney DM Clinical characteristics of aortic aneurysm and dissection as a cause of sudden death in outpatients Am J Emerg Med 2008 26 9 1042 1046 10.1016/j.ajem.2007.12.014 19091267 PMC2605727

[B8] Manthous CA Avoiding circulatory complications during endotracheal intubation and initiation of positive pressure ventilation J Emerg Med 2010 38 5 622 631 10.1016/j.jemermed.2009.01.018 19464138

[B9] Pitts S Kellermann AL Hyperventilation during cardiac arrest Lancet 2004 364 9431 313 315 10.1016/S0140-6736(04)16740-8 15276374

[B10] Aufderheide TP Sigurdsson G Pirrallo RG Yannopoulos D McKnite S von Briesen C Sparks CW Conrad CJ Provo TA Lurie KG Hyperventilation-induced hypotension during cardiopulmonary resuscitation Circulation 2004 109 16 1960 1965 10.1161/01.CIR.0000126594.79136.61 15066941

[B11] Snyder HS Lack of a tachycardic response to hypotension with ruptured ectopic pregnancy Am J Emerg Med 1990 8 1 23 26 10.1016/0735-6757(90)90288-B 2293828

[B12] Whitehead SJ Berg CJ Chang J Pregnancy-related mortality due to cardiomyopathy: United States, 1991-1997 Obstet Gynecol 2003 102 6 1326 1331 10.1016/j.obstetgynecol.2003.08.009 14662222

[B13] Adamson DL Nelson-Piercy C Managing palpitations and arrhythmias during pregnancy Heart 2007 93 12 1630 1636 18003696 10.1136/hrt.2006.098822 PMC2095764

[B14] Siu SC Sermer M Colman JM Alvarez AN Mercier LA Morton BC Kells CM Bergin ML Kiess MC Marcotte F Taylor DA Gordon EP Spears JC Tam JW Amankwah KS Smallhorn JF Farine D Sorensen S Cardiac Disease in Pregnancy (CARPREG) Investigators Prospective multicenter study of pregnancy outcomes in women with heart disease Circulation 2001 104 5 515 521 10.1161/hc3001.093437 11479246

[B15] Tan HL Lie KI Treatment of tachyarrhythmias during pregnancy and lactation Eur Heart J 2001 22 6 458 464 10.1053/euhj.2000.2130 11237540

[B16] Kiss G Arvieux CC Remarks on guidelines ERC 2000: cardiac arrest associated with pregnancy Resuscitation 2004 61 3 367 10.1016/j.resuscitation.2004.01.028 15172719

[B17] Vanden Hoek TL Morrison LJ Shuster M Donnino M Sinz E Lavonas EJ Jeejeebhoy FM Gabrielli A Part 12: cardiac arrest in special situations: 2010 American Heart Association Guidelines for Cardiopulmonary Resuscitation and Emergency Cardiovascular Care Circulation 2010 122 18 Suppl 3 S829 S861 20956228 10.1161/CIRCULATIONAHA.110.971069

[B18] Katz V Balderston K DeFreest M Perimortem cesarean delivery: were our assumptions correct? Am J Obstet Gynecol 2005 192 1916 1920 10.1016/j.ajog.2005.02.038 15970850

[B19] Jeejeebhoy FM Zelop CM Windrim R Carvalho JC Dorian P Morrison LJ Management of cardiac arrest in pregnancy: a systematic review Resuscitation 2011 82 7 801 809 10.1016/j.resuscitation.2011.01.028 21549495

[B20] Dijkman A Huisman CM Smit M Schutte JM Zwart JJ van Roosmalen JJ Oepkes D Cardiac arrest in pregnancy: increasing use of perimortem caesarean section due to emergency skills training BJOG 2010 117 3 282 287 10.1111/j.1471-0528.2009.02461.x 20078586

[B21] Flippin A Hendricks SK Reichman EF, Simon RR Perimortem cesarean section Emergency Medicine Procedures 2004 New York: McGraw-Hill 1070 1078

[B22] Valenzuela TD Kern KB Clark LL Berg RA Berg MD Berg DD Hilwig RW Otto CW Newburn D Ewy GA Interruptions of chest compressions during emergency medical systems resuscitation Circulation 2005 112 9 1259 1265 10.1161/CIRCULATIONAHA.105.537282 16116053

[B23] Neumar RW Otto CW Link MS Kronick SL Shuster M Callaway CW Kudenchuk PJ Ornato JP McNally B Silvers SM Passman RS White RD Hess EP Tang W Davis D Sinz E Morrison LJ Part 8: adult advanced cardiovascular life support: 2010 American Heart Association Guidelines for Cardiopulmonary Resuscitation and Emergency Cardiovascular Care Circulation 2010 122 18 Suppl 3 S729 S767 20956224 10.1161/CIRCULATIONAHA.110.970988

[B24] Abella BS Sandbo N Vassilatos P Alvarado JP O'Hearn N Wigder HN Hoffman P Tynus K Vanden Hoek TL Becker LB Chest compression rates during cardiopulmonary resuscitation are suboptimal: a prospective study during in-hospital cardiac arrest Circulation 2005 111 4 428 434 10.1161/01.CIR.0000153811.84257.59 15687130

[B25] Kellum MJ Kennedy KW Barney R Keilhauer FA Bellino M Zuercher M Ewy GA Cardiocerebral resuscitation improves neurologically intact survival of patients with out-of-hospital cardiac arrest Ann Emerg Med 2008 52 3 244 252 10.1016/j.annemergmed.2008.02.006 18374452

[B26] Ewy GA Kern KB Recent advances in cardiopulmonary resuscitation: cardiocerebral resuscitation J Am Coll Cardiol 2009 53 2 149 157 10.1016/j.jacc.2008.05.066 19130982

[B27] Bernard SA Gray TW Buist MD Jones BM Silvester W Gutteridge G Smith K Treatment of comatose survivors of out-of-hospital cardiac arrest with induced hypothermia N Engl J Med 2002 346 8 557 563 10.1056/NEJMoa003289 11856794

[B28] Hypothermia after Cardiac Arrest Study Group Mild therapeutic hypothermia to improve the neurologic outcome after cardiac arrest N Engl J Med 2002 346 8 549 556 11856793 10.1056/NEJMoa012689

[B29] Nolan JP Morley PT Vanden Hoek TL Hickey RW Kloeck WG Billi J Böttiger BW Morley PT Nolan JP Okada K Reyes C Shuster M Steen PA Weil MH Wenzel V Hickey RW Carli P Vanden Hoek TL Atkins D International Liaison Committee on Resuscitation Therapeutic hypothermia after cardiac arrest: an advisory statement by the advanced life support task force of the International Liaison Committee on Resuscitation Circulation 2003 108 1 118 121 10.1161/01.CIR.0000079019.02601.90 12847056

[B30] 2005 AHA Guidelines for CPR and ECC Circulation 2005 112 Suppl I IV-84 IV-88 10.1161/CIRCULATIONAHA.105.170809 16314349

[B31] Dumas F Grimaldi D Zuber B Fichet J Charpentier J Pène F Vivien B Varenne O Carli P Jouven X Empana JP Cariou A Is hypothermia after cardiac arrest effective in both shockable and nonshockable patients?: Insights from a large registry Circulation 2011 123 8 877 886 10.1161/CIRCULATIONAHA.110.987347 21321156

[B32] Johnson S Henderson SO Myth: the Trendelenburg position improves circulation in cases of shock CJEM 2004 6 1 48 49 17433146 10.1017/s1481803500008915

[B33] Link MS Atkins DL Passman RS Halperin HR Samson RA White RD Cudnik MT Berg MD Kudenchuk PJ Kerber RE Part 6: electrical therapies: automated external defibrillators, defibrillation, cardioversion, and pacing: 2010 American Heart Association Guidelines for Cardiopulmonary Resuscitation and Emergency Cardiovascular Care Circulation 2010 122 18 Suppl 3 S706 S719 20956222 10.1161/CIRCULATIONAHA.110.970954

[B34] Lloyd MS Heeke B Walter PF Hands-on defibrillation: An analysis of electrical current flow through rescuers in direct contact with patients during biphasic external defibrillation Circulation 2008 117 2510 2514 10.1161/CIRCULATIONAHA.107.763011 18458166

[B35] Harper RJ Roberts JR, Hedges JR, et al Pericardiocentesis Clinical Procedures in Emergency Medicine 2010 5 Saunders, Philadelphia

[B36] Meyers DG Bagin RG Levene JF Electrocardiographic changes in pericardial effusion Chest 1993 104 5 1422 1426 10.1378/chest.104.5.1422 8222799

[B37] Ho AM Graham CA Ng CS Yeung JH Dion PW Critchley LA Karmakar MK Timing of tracheal intubation in traumatic cardiac tamponade: a word of caution Resuscitation 2009 80 2 272 274 10.1016/j.resuscitation.2008.09.021 19059695

[B38] Fengler B Brady W Fibrinolytic therapy in pulmonary embolism: an evidence-based treatment algorithm Am J Emerg Med 2009 27 1 84 95 10.1016/j.ajem.2007.10.021 19041539

[B39] Casazza F Bongarzoni A Capozi A Agostoni O Regional right ventricular dysfunction in acute pulmonary embolism and right ventricular infarction Eur J Echocardiogr 2005 6 1 11 14 10.1016/j.euje.2004.06.002 15664548

[B40] Kosuge M Kimura K Ishikawa T Ebina T Hibi K Kusama I Nakachi T Endo M Komura N Umemura S Electrocardiographic differentiation between acute pulmonary embolism and acute coronary syndromes on the basis of negative T waves Am J Cardiol 2007 99 6 817 821 10.1016/j.amjcard.2006.10.043 17350373

